# Health-Related Quality-of-Life Utility Values in Adults With Late-Onset Pompe Disease: Analyses of EQ-5D Data From the PROPEL Clinical Trial

**DOI:** 10.36469/001c.121928

**Published:** 2024-09-18

**Authors:** Alasdair MacCulloch, Alison Griffiths, Neil Johnson, Simon Shohet

**Affiliations:** 1 Amicus Therapeutics UK Ltd, Marlow, UK; 2 Research Economics, Solihull, UK

**Keywords:** EQ-5D, health-related quality of life, Pompe disease, PROPEL, Pombiliti®/Opfolda® (cipaglucosidase alfa + miglustat), HRQoL

## Abstract

**Background:** Pompe disease is a rare lysosomal storage disorder, leading to accumulation of glycogen characterized by muscle weakness, fatigue, pain, and, in the longer term, a requirement for ventilatory and ambulatory support, and early mortality if untreated. Clinical evidence suggests that enzyme replacement therapy improves health outcomes for adults with late-onset Pompe disease (LOPD). PROPEL was a Phase 3, double-blind, randomized controlled trial, which evaluated cipaglucosidase alfa plus miglustat, vs alglucosidase alfa plus placebo in 123 adult patients with LOPD (clinicaltrials.gov: NCT03729362). **Objectives:** To analyze EQ-5D health-related quality of life (HRQoL) utility data from PROPEL. **Methods:** Multilevel modeling techniques (mixed regression methods) were used to analyze PROPEL EQ-5D-3L estimates and predict utility values for 7 health states previously identified in an economic evaluation for LOPD. In PROPEL, EQ-5D-5L values were assessed at screening and at weeks 12, 26, 38, and 52. EQ-5D-5L utility values were mapped to EQ-5D-3L values using the van Hout algorithm as recommended by the EuroQoL and the National Institute of Health and Care Excellence position statement at time of analysis. UK population tariffs were applied for all EQ-5D utility valuations. Utility values were predicted according to 6-minute walk distance (6MWD) and percent predicted sitting forced vital capacity. **Results:** The mixed model predicted that EQ-5D-3L utility values for patients who could walk >75 m with LOPD ranged between 0.55 and 0.67 according to patient 6MWD and respiratory function. In this analysis, patients with a 6MWD ≤75 m, consistent with a health state requiring wheelchair support in the economic analysis, had a predicted utility value of 0.49. There were few patients in PROPEL who could walk ≤75 m at any time point in the study, hence, these utility estimates should be interpreted with caution. EQ-5D-3L utility estimates from PROPEL were consistent with previously reported EQ-5D-3L values in LOPD. **Conclusions:** Overall, the results from our analysis indicate that important HRQoL losses are associated with reductions in mobility and respiratory function for patients with Pompe disease. The study provides important evidence of HRQoL utility values for patients with advanced LOPD, a population for whom published data are limited.

## INTRODUCTION

Pompe disease is a rare genetic lysosomal storage disorder associated with complete or partial loss of endogenous acid α-glucosidase (GAA) activity, which results in an accumulation of glycogen in the body.^1–3^ It is characterized by a progressive loss of muscle function resulting in weakness, fatigue, pain, exercise intolerance and, in the longer term, a requirement for ventilatory and ambulatory support.^1–3^ The primary cause of death in patients with Pompe disease is respiratory failure, occurring in approximately 70% of diagnosed patients.^4,5^ Pompe disease is associated with significant health-related quality of life (HRQoL) losses over a patient’s lifetime due to the severity of symptoms.^6^ There are 2 primary subtypes of Pompe disease: infantile-onset Pompe disease, which presents at a very young age, and late-onset Pompe disease (LOPD), which presents in children, juveniles, and adults. Prevalence estimates are rarely published and vary widely within Europe; a study in Belgium suggested the prevalence of LOPD was 1 in 57 000, while a study in The Netherlands reported estimates of 1 in 250 000.^5,7^

Current standard-of-care treatment for patients with LOPD is enzyme replacement therapy (ERT) with recombinant human acid α-glucosidase (rhGAA), which is designed to improve lysosomal glycogen degradation and slow disease progression.^8^ The first approved ERT was alglucosidase alfa (Myozyme^®^).^9^ It is estimated that 24% to 30% of patients do not respond to alglucosidase alfa as measured according to 6-minute walk distance (6MWD) and percent predicted forced vital capacity (% predicted FVC).^10^ Nevertheless, in those who do initially respond, current evidence indicates there can also be a progressive deterioration of function over time. In a 2021 study by Gutschmidt et al,^11^ patients with 3 or more years of alglucosidase alfa treatment at study baseline demonstrated a 14.9% decline in % predicted FVC over the next 10 years of treatment with alglucosidase alfa; over the same time period, the percentage of patients requiring ventilation increased by 33%.^11^ Consequently, there remains a significant need for alternative treatments for Pompe disease that are effective and well tolerated.

Cipaglucosidase alfa (cipa) is a novel (Chinese hamster ovary cell–derived) rhGAA with enhanced glycosylation for improved cellular uptake and retained capacity for processing into the most active form of the enzyme. Cipa is administered in combination with miglustat (mig; cipa + mig; Pombiliti^®^/Opfolda^®^), an orally administered small-molecule stabilizer of cipa, which acts in the circulation so that catalytic activity is maintained upon uptake to the muscles.^12,13^ Cipa + mig has recently received market authorization from the US Food and Drug Administration, the European Medicines Agency, and the Medicines and Healthcare products Regulatory Agency in the United Kingdom following positive results from the pivotal PROPEL clinical trial (clinicaltrials.gov: NCT03729362).^3,14–16^

PROPEL was a multicenter, international, Phase 3 randomized controlled trial designed to evaluate cipa + mig vs alglucosidase alfa plus placebo (alg + pbo) in 123 adult patients with LOPD.^3^ In PROPEL, cipa + mig was associated with clinically meaningful improvements in key motor and respiratory outcomes compared with standard-of-care ERT.^3^ The PROPEL clinical study also indicated numeric improvements for cipa + mig vs alg + pbo in most patient-reported outcomes.^3^

The overall aim of this study was to predict EQ-5D utility estimates for health states associated with LOPD, which have been defined according to patient mobility (6MWD) and respiratory function (% predicted FVC). It is anticipated that the results from these analyses will improve the evidence base for Pompe disease currently available to healthcare decision makers.

## METHODS

### PROPEL

In PROPEL, patients received 20 mg/kg of cipaglucosidase alfa via intravenous (IV) infusion plus oral miglustat (195 mg or 260 mg for body weights <50 kg, ≥50 kg, respectively) or alglucosidase alfa (20 mg/kg IV) plus matching placebo, biweekly for 52 weeks. Randomization was stratified according to baseline 6MWD from ≥75 m to <150 m, ≥150 m to <400 m, or ≥400 m and ERT status (naïve vs experienced). The primary objective was to assess the efficacy of cipa + mig vs alg + pbo using the 6-minute walk test. A key secondary objective was to assess the efficacy of cipa + mig vs alg + pbo on pulmonary function, as measured by % predicted FVC. Patient-reported outcomes data were collected using 4 instruments: EQ-5D-5L, Patient-Reported Outcomes Measurement Information System, Rasch-Built Pompe-Specific Activity Scale, and Subject’s Global Impression of Change. The full study design and results for PROPEL have been previously reported.^3^

### EQ-5D

The EQ-5D-5L is a generic instrument that assesses disease severity across 5 dimensions of a patient’s HRQoL (mobility, self-care, usual activities, pain/discomfort, and anxiety/depression) and can be used to generate health-related utility values. Utility values typically represent patients’ quality of life on a scale where 0 represents death and 1 represents full health (although negative values are feasible).^5,17^ In an economic evaluation, utility values are used to “quality adjust” patient survival time and reflect important differences in the quality of life for surviving patients.

EQ-5D-5L data were collected during PROPEL at repeated intervals (at screening and weeks 12, 26, 38, and 52). EQ-5D-5L is the updated version of the EQ-5D-3L questionnaire in which, for each of the 5 domains, patients are required to select 1 of 5 possible responses (no problems, slight problems, moderate problems, severe problems, and extreme problems).^17^ The earlier EQ-5D-3L questionnaire required patients to select 1 of 3 possible responses for each domain (no problems, moderate problems, extreme problems). The National Institute for Health and Care Excellence (NICE) technology assessment guidelines recommend using EQ-5D-3L values in UK health technology submissions due to issues with cross-validation of the EQ-5D-5L questionnaire.^6,18^ In line with EuroQoL recommendations and NICE recommendations at the time of the analysis, PROPEL EQ-5D-3L utility values were mapped from EQ-5D-5L domain scores using the van Hout crosswalk algorithm.^19^ Our base-case analyses reported EQ-5D-3L utility estimates, while EQ-5D-5L results were considered in sensitivity analyses and were reported in supplementary material. The analysis took a UK perspective and applied UK population tariffs for all EQ-5D utility valuations.

### Health States

We used PROPEL data to derive utility values for 7 health states, consistent with the health states used in recently published cost-effectiveness analyses in LOPD and were defined using a combination of patient mobility and respiratory function as follows^4–9^:

1. No wheelchair use or respiratory support
2. Intermittent mobility support
3. Intermittent respiratory support (noninvasive ventilation)
4. Intermittent mobility support and intermittent respiratory support (noninvasive ventilation)
5. Wheelchair-dependent
6. Wheelchair-dependent and intermittent respiratory support (noninvasive ventilation)
7. Wheelchair and respiratory support-dependent (invasive ventilation)

The thresholds for 6MWD and % predicted FVC, which determine mobility and respiratory function in the cost-effectiveness analysis (and hence in this study), were derived with the input from 3 clinical experts and validated using opinion from a further 2 experts (**Table 1**). It is noted that the PROPEL dataset was representative of the first 4 health states of interest, patients who required wheelchair support were not eligible for randomization during the PROPEL trial. Due to this limitation, we have derived utility estimates for more severe health states, in which both respiratory and wheelchair support were required, using a series of assumptions and a multiplicative approach consistent with NICE guidance.^7,20^

**Table 1.** Health State Definitions: Cost-Utility Analysis

**Support**	**Maximum Threshold**	**Source**
Intermittent mobility support (max m in 6MWD)	250	UK clinical opinion
Wheelchair-dependent (max m in 6MWD)	75	
Intermittent respiratory support (max % predicted FVC)	40	Assumption
Respiratory support-dependent (max % predicted FVC)	30	UK clinical opinion

Abbreviations: 6MWD, 6-minute walk distance; FVC, forced vital capacity; max, maximum.

Utility values for patients in the health state for intermittent mobility support and intermittent respiratory support (noninvasive ventilation) were based on the ratio of patients requiring intermittent respiratory support vs no support, applied to utility estimates for intermittent mobility support. To estimate the potential severity of utility loss associated with the combination of wheelchair use and noninvasive respiratory support, the multiplier was based on the relative utility loss for wheelchair use vs full mobility, applied to the health state for intermittent mobility and respiratory support. Due to the severity of a health state associated with wheelchair use and invasive ventilation, the same approach was used with the multiplier raised to the second power.

### Statistical Analyses

Summary statistics based on EQ-5D-3L and EQ-5D-5L estimates were prepared. The EQ-5D datasets were longitudinal, repeated measures datasets with multiple observations available for each individual patient, hence, multilevel modeling techniques (also known as mixed models) were considered for the primary analysis. In the presence of clustering, mixed models provide a more reliable estimate of predicted outcomes compared to conventional regression methods. Estimates of the intraclass correlation coefficient and a likelihood ratio test comparing a mixed model to a standard linear model were used to establish the most suitable approach to analyzing PROPEL data. In our final analysis, we assumed that EQ-5D scores within each patient were correlated and a random intercept mixed model was developed.

The mixed regression model was developed using a multivariable regression technique. The base-case outcome variable was EQ-5D-3L utility scores. The explanatory variables considered for potential inclusion were based on the clinical study protocol and included treatment, baseline EQ-5D-3L (EQ-5D-5L for the sensitivity analysis), baseline values of key clinical outcomes (% predicted FVC, 6MWD), prior ERT experience (yes, no), duration of prior ERT (years), baseline use of mobility devices (yes, no) and history of falls (yes, no), as well as patient characteristics known to be associated with HRQoL (age, sex, body mass index). Variables were selected using backward stepwise elimination and corroborated using forward selection. The variables finally included in the regression model were those variables that demonstrated evidence of an important association with HRQoL, which was based on the direction, magnitude, and significance of the effect on utility outcomes (*P* < .05), as well as the impact on other explanatory variables. Correlation matrices were reviewed for evidence of potential collinearity between explanatory variables.

All analyses were conducted in STATA statistical software: Release 14 (StataCorp LP).

## RESULTS

### Baseline Characteristics

In total, 123 adults were randomized in PROPEL. The majority of patients were ERT-experienced (76% cipa + mig group, 79% alg + pbo group). As reported previously,^3^ there were some slight numeric differences in baseline characteristics between the cipa + mig and alg + pbo groups. Notably, patients treated with cipa + mig, compared with those receiving alg + pbo, were more likely to be female (58% vs 47%), be slightly older (aged 48 vs 45 years),^3^ and have slightly poorer baseline HRQoL (EQ-5D-3L value at baseline: 0.634 vs 0.669; **Supplementary Table S1**).

### EQ-5D Outcome Data

EQ-5D data were available for 122/123 patients across all 5 data collection points. It is noted that 1 patient in the alg + pbo group was not included in final estimates for the primary and secondary outcomes in the main PROPEL trial due to evidence of reporting bias. This patient was also excluded from our EQ-5D analysis. A normal probability plot demonstrated some evidence of non-normality; however, most data points lay over the range between 0.3 and 0.9, and the non-normality of the data was not considered extreme (see **Supplementary Figure S1)**. Transformation of the outcome variable was not undertaken since residuals from the final analysis appeared approximately normally distributed.

### Initial Analyses

A log-likelihood ratio test comparing a standard linear model with linear mixed model showed statistical significance (*P* < .001), suggesting a multilevel regression model was preferable to a generalized linear model. The intraclass correlation coefficient was also greater than 0.5 for cluster variables, which suggested evidence of important correlation between measurements from the same patient and further evidence that a multilevel regression model would be preferred to a generalized linear model. Univariable, random effects, mixed regression analyses considering the effect of each explanatory variable individually indicated that patient age, sex, mobility issues, % predicted FVC, 6MWD were all potentially associated with patient HRQoL.

### Multivariable Mixed Regression

Multivariable analyses indicated that only 6MWD and sex were significant predictors of HRQoL. Female patients appeared to have poorer HRQoL than male patients, a trend that has been previously reported in other large HRQoL studies and is not considered unique to PROPEL data.^21,22^ While % predicted FVC was also independently associated with patient HRQoL in univariable analyses (*P* = .03), additional exploratory analysis indicated a strong correlation between % predicted FVC and 6MWD (**Supplementary Figure S2**). The introduction of both 6MWD and % predicted FVC into the multivariable regression resulted in large changes in the parameter estimates for explanatory variables, suggestive of collinearity. Furthermore, when assessed using a multivariable model that included both % predicted FVC and 6MWD, % predicted FVC was no longer a significant predictor of HRQoL. Overall, 6MWD appeared to be a much stronger predictor of HRQoL compared with % predicted FVC; hence, 6MWD was retained in the base-case regression model (**Table 2**). We presented an equation without sex as a potential predictor since differences in HRQoL outcomes by sex would not typically be considered in an economic evaluation of a new intervention due to equity considerations. A risk equation that includes sex has been presented in supplementary materials (**Supplementary Table S2**).

**Table 2.** EQ-5D-3L Mixed Regression Model: 6MWD

**Category**	**Coef.**	**SE**	**z**	***P* > z**	**95% LCI**	**95% UCI**
6MWD	0.001	0.000	5.500	0.000	0.000	0.001
Constant	0.470	0.036	13.050	0.000	0.399	0.540

Abbreviations: 6MWD, 6-minute walk distance; coef, coefficient; LCI, lower bound confidence interval; SE, standard error; UCI, upper bound confidence interval.

### Health State Predictions

The results from the base-case mixed regression analysis indicate that EQ-5D-3L utility values for non-wheelchair-dependent patients were predicted as 0.55 (6MWD >75 to ≤250 m) and 0.67 (6MWD >250 m) (**Table 3**). In the mixed regression model, patients who could walk ≤75 m distance were predicted to have a utility value of 0.49. The base-case algorithm excluded respiratory function as a predictor of HRQoL utility; hence, utility predictions for health states purely associated with a deterioration in respiratory function could not be obtained from the base-case equation. To address this limitation, a separate risk equation was developed based purely on % predicted FVC (**Table 4**). The predicted utility value for patients requiring intermittent respiratory support (% predicted FVC >30 to ≤40%) was 0.61 (**Table 5**). The derived composite utility values derived using assumptions regarding the potential utility loss for more severe health states indicated that patient utility may range between 0.19 and 0.37 (**Table 6**).

**Table 3.** EQ-5D-3L Predicted Utility Values by 6MWD

**Category**	**6MWD (m)**		
	**≤75**	**>75 to ≤250**	**>250^a^**
EQ-5D-3L utility value	0.49	0.55	0.67

Note: Utility values estimated for category midpoints.

^a^Utility predictions based on mean 6MWD for patients with a 6MWD >250 m (mean: 406 m). Abbreviation: 6MWD, 6-minute walk distance.

**Table 4.** EQ-5D-3L Mixed Regression Model: % Predicted FVC

**Category**	**Coef.**	**SE**	**z**	***P* > z**	**95% LCI**	**95% UCI**
% predicted FVC	0.0012	0.0006	2.1600	0.0310	0.0001	0.0024
Constant	0.5681	0.0424	13.4000	0.0000	0.4850	0.6512

Abbreviations: coef, coefficient; FVC, forced vital capacity; LCI, lower bound confidence interval; SE, standard error; UCI, upper bound confidence interval.

**Table 5.** EQ-5D-3L Predicted Utility Values by % Predicted FVC

**Category**		**% Predicted FVC**	
	≤**30**	**>30 to ≤40**	**>40^a^**
EQ-5D-3L utility value	0.59	0.61	0.66

Note: Utility values estimated for category midpoints.

^a^Utility predictions based on mean % predicted FVC for patients with a predicted FVC >40% (mean: 72.1). Abbreviation: FVC, forced vital capacity.

**Table 6.** PROPEL vs Published Health State Utility Values in Pompe Disease

**Health State**	**PROPEL EQ-5D-3L**	**PROPEL EQ-5D-5L**	**Avalglucosidase alfa (Nexviadyme™) NICE TA821^23^**	**Vignette Study EQ-5D-5L^6^**	**Vignette Study TTO^6^**
No wheelchair use or respiratory support (>15 y)	0.67	0.75	0.65	0.61	0.75
Intermittent mobility support	0.55	0.62	–	0.43	0.61
Intermittent respiratory support (noninvasive ventilation)	0.62	0.69	0.61	0.36	0.56
Intermittent mobility support and intermittent respiratory support (noninvasive ventilation)	**0.51**	**0.57**	0.55	0.29	0.41
Wheelchair-dependent	0.49	0.55	0.50	0.11	0.34
Wheelchair-dependent and intermittent respiratory support (noninvasive ventilation)	**0.37**	**0.41**	0.40	0.08	0.24
Wheelchair and respiratory support-dependent (invasive ventilation)	**0.19**	**0.23**	–	−0.08	0.13

Bolded values: Composite utility values derived from combination of scores from other health states. Abbreviations: NICE, National Institute of Health and Care Excellence; TTO, time trade-off.

### Sensitivity Analysis: EQ-5D-5L

Overall, the results for analyses based on the EQ-5D-5L data showed comparable trends to those based on EQ-5D-3L values, although overall the EQ-5D-5L utility estimates were higher than those derived based on EQ-5D-3L estimates. The analyses of EQ-5D-5L data suggested that utility values ranged between 0.62 and 0.75 for non-wheelchair-dependent patients with LOPD, while those who could walk ≤75 m were predicted to have a utility value of 0.55 (**Supplementary Table S3** and **Supplementary Table S4**).

## DISCUSSION

The PROPEL trial demonstrated that cipa + mig was associated with clinically meaningful improvements in key motor and respiratory outcomes compared with alg + pbo in adult patients with LOPD.^3^ The objective of this study was to analyze EQ-5D data from PROPEL according to treatment, key clinical outcomes (% predicted FVC, 6MWD) and other potential predictors of HRQoL to provide utility estimates for key health states previously associated with Pompe disease. In the base-case analysis we have developed a mixed model using longitudinal EQ-5D-3L estimates from PROPEL. This model indicated only 6MWD (and sex) were strongly associated with patient HRQoL utility scores. The EQ-5D-3L utility values for non-wheelchair-dependent LOPD patients ranged from 0.55 (6MWD >75 to ≤250 m) to 0.67 (6MWD >250 m), while patients with a 6MWD ≤75 m, who were likely to require wheelchair support, were estimated to have a utility value of 0.49.

The current study has used the van Hout crosswalk algorithm to map EQ-5D-5L values to EQ-5D-3L as recommended by EuroQoL and in keeping with the NICE EQ-5D-5L position statement at the time of the analysis. It is acknowledged that the current NICE health technology assessment manual recommends an alternative mapping function by Hernandez-Alava et al.^18,24^ Comparisons of the 2 approaches suggest only small differences in goodness-of-fit, and we do not anticipate that the use of the Hernandez-Alava approach would alter conclusions from this study.^23,24^

The limitations of this study are primarily associated with the data available, which were constrained by the rarity of the disease. PROPEL, although the largest randomized controlled trial to date in Pompe disease, was a relatively small study (n = 123), and the eligibility criteria required all patients to be ambulatory and requiring ≤6 hours/day ventilatory support. This meant that few patients were in the later stages of disease progression, which made predictions for more health states associated with more severe disease challenging. It is acknowledged that the study may not have been powered specifically to adequately characterize the effect of all variables that impact patient HRQoL, reflecting this common challenge in rare disease trials. Multivariable analyses indicated that when both % predicted FVC and 6MWD were included in the regression model, % predicted FVC was no longer a significant predictor of HRQoL and there was evidence of collinearity, hence % predicted FVC was not included in the final equation. Thus, to provide utility values for health states associated with respiratory function, a separate risk equation was developed based purely on % predicted FVC as an explanatory variable. It is noted that the algorithm was based on the same data as the algorithm for 6MWD, hence, the combination of these estimates may result in some overestimation of the utility losses associated with composite health states, which include reductions in both respiratory function and patient mobility.

Nevertheless, despite these limitations, this study provides important evidence of HRQoL utility values across the spectrum of health states for patients with LOPD, a population for whom there are currently limited published data. The utility estimates in this study are highly consistent with estimates of EQ-5D utility values from the small number of previously published studies in LOPD.^6,8^ Notably, a recent NICE health technology assessment of avalglucosidase alfa (Nexviadyme™) in Pompe disease (TA821) indicated that non-wheelchair-dependent patients may have an EQ-5D-3L utility value between 0.55 and 0.65 according to patient mobility and respiratory support requirements (**Table 6**).^8^ The closeness of these estimates to EQ-5D 3L values we have derived from PROPEL suggests cross-validity between studies.

It is acknowledged that a recent vignette study by Hubig et al reported lower utility EQ-5D-5L values for LOPD patients than PROPEL. This difference may be explained by differences in the methods of utility estimation between the vignette study and the PROPEL clinical trial.^3,6^ The vignette study created a series of health state descriptions based on interviews with patients with LOPD and UK clinical experts. One hundred members of the general public were asked to imagine they were in the vignette health states and value their HRQoL utility value using 3 elicitation methods: EQ-5D-5L, visual analog scale, and time trade-off (TTO). PROPEL utility values were based on EQ-5D-5L questionnaires completed by 123 adults with LOPD. It has been noted elsewhere that individuals in the general population are likely to overstate the disability in a given health state compared with those who have been suffering from the disease and adapting to their condition over time. Furthermore, in the vignette study, there was some inconsistency between utility values estimated from the 3 different elicitation methods: notably, EQ-5D-5L values were much lower than TTO values. The vignette TTO values were more consistent with EQ-5D-3L results presented in this work than the vignette EQ-5D-5L values. It was unclear why the vignette EQ-5D-5L results were low, but this trend has been seen in previous research.^22^ These findings provide an interesting insight into the potential challenges associated with estimating utilities in rare diseases when utility data are not captured within the clinical study itself.

Very few patients were in the late stages of LOPD progression in the PROPEL trial; hence, predictions from our study for patients in more severe health states data may not be reliable and should be interpreted with appropriate caution. In particular, it is noted that no patients who were wheelchair-dependent were using invasive ventilation in PROPEL. The reported utility value has been derived using strong assumptions regarding the relative loss in utility for patients requiring invasive ventilation. These assumptions merit further clinical validation, and the values derived are difficult to cross-validate, since utility values for severe LOPD health states have been rarely reported in the published literature. The vignette study by Hubig et al suggested that EQ-5D-5L patient utility for this health state would be less than 0 (ie, this health state was valued as worse than death), although, in the NICE appraisal of cipa + mig, the NICE Evidence Assessment Group noted that it is rare to apply utility values that are significantly below 0.50, and rarer still to assign negative utility values.^4^

It is noted that mixed models cannot consider any bias associated with data not missing at random. Informative censoring may occur when patients in poorer health do not provide HRQoL responses. While this may be an important consideration in Pompe disease due to the severity of symptoms, given the relatively earlier stage of disease progression of patients in PROPEL and high completion rate, it is not considered an issue in this study.

## CONCLUSION

We have estimated utility values for 7 health states, which have been defined according to patient respiratory function and mobility commonly associated with LOPD. Overall, the results from our analysis indicate that important HRQoL losses are associated with reductions in mobility and respiratory function for patients with Pompe disease. The study provides important evidence of HRQoL utility values for patients with LOPD, including advanced LOPD, a population for whom there are currently limited published data.

### Disclosures

A.M., N.J. and S.S. are employees of Amicus Therapeutics UK, Ltd and hold stock in Amicus Therapeutics, Inc. A.G. is employed by Research Economics. Research Economics received funding from Amicus Therapeutics UK, Ltd in relation to this study.

## Supplementary Material

Online Supplementary Material
