# Supplementary material for: Health-Related Quality-of-Life Utility Values in Adults With Late-Onset Pompe Disease: Analyses of EQ-5D Data From the PROPEL Clinical Trial
Source: J Health Econ Outcomes Res. 2024 Sep 18;11(2):80–5. doi: 10.36469/001c.121928 (PMC11420789; doi:10.36469/001c.121928)
Supplement: Online Supplementary Material [file jheor_2024_11_2_121928_246397.pdf]

Journal of Health Economics and Outcomes Research

Online Supplementary Material

Health-Related Quality of Life Utility Values in Adults Living with Late-Onset Pompe disease: Analyses of EQ-5D Data from the PROPEL Clinical Trial. *JHEOR*. 2024;11(2):80-85. doi:10.36469/jheor.2024.121928

Figure S1: Normal Probability Plot: EQ-5D-3L Utility Values

Figure S2: Correlation Between % Predicted FVC and 6MWD

Table S1: Further Baseline Characteristics PROPEL Phase 3 Trial

Table S2: EQ-5D-3L Mixed Regression Model: 6MWD (Centered on Mean)

Table S3: EQ-5D-5L Mixed Regression Model: 6MWD

Table S4: EQ-5D-5L Predicted Utility Values by 6MWD

This supplementary material has been provided by the authors to give readers additional information about their work.

This is an open-access article distributed under the terms of the Creative Commons Attribution 4.0 International License (CCBY-4.0). View this license's legal deed at http://creativecommons.org/licenses/by/4.0 and legal code at http://creativecommons.org/licenses/by/4.0/legalcode for more information.

Figure S1. Normal Probability Plot: EQ-5D-3L Utility Values

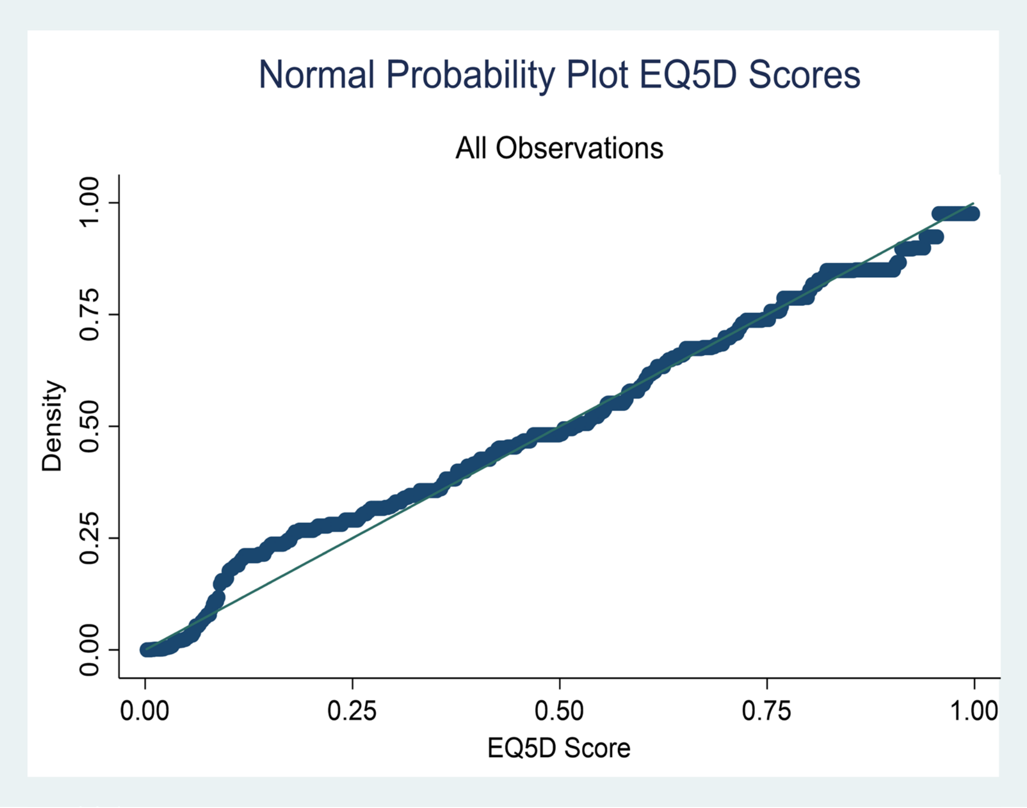

Figure S2. Correlation Between % Predicted FVC and 6MWD

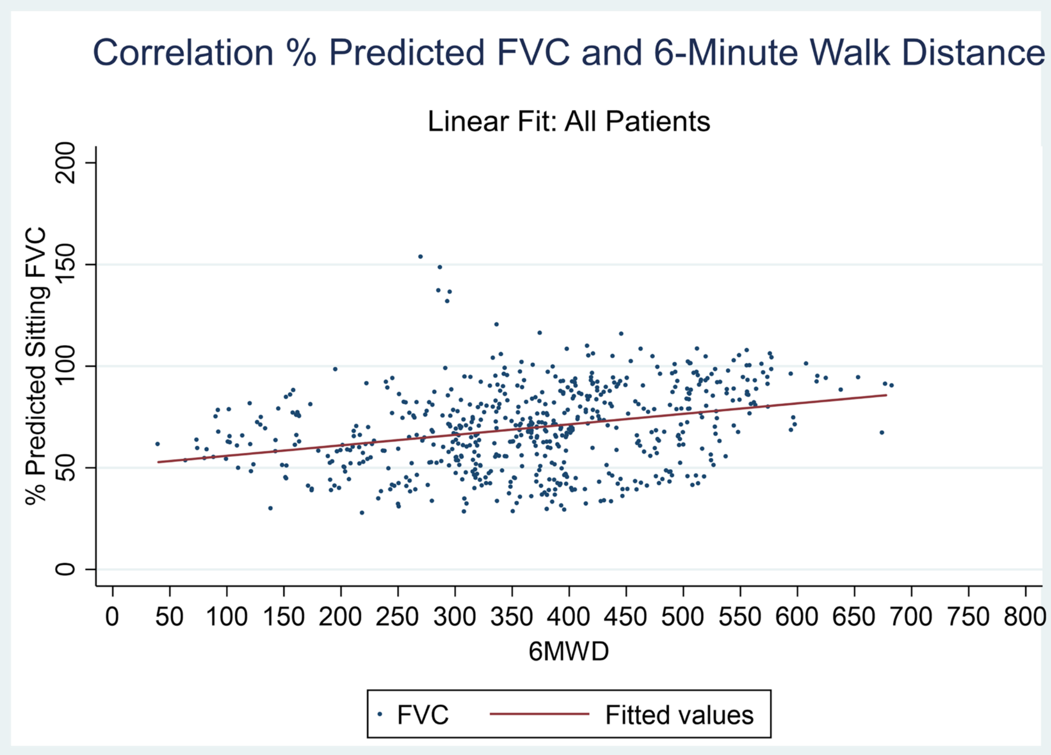

Abbreviations: 6MWD, 6-minute walk distance; FVC, forced vital capacity.

Table S1. Further Baseline Characteristics PROPEL Phase 3 Trial

Baseline Variable, Mean (SD)	Cipaglucosidase alfa + Miglustat (n = 85)	Alglucosidase alfa + Placebo (n = 38)
BMI, kg/m^2	24.7 (4.5)	26.7 (7.3)
EQ-5D-5L	0.72 (0.18)	0.75 (0.16)
EQ-5D-3L[a]	0.63 (0.17)	0.67 (0.14)

[a]EQ-5D-3L utility values were mapped from EQ-5D-5L domain scores using the van Hout crosswalk algorithm (van Hout B, Janssen MF, Feng YS, et al. *Value Health.* 2012;15(5):708-715).
Abbreviations: BMI, body mass index; SD, standard deviation.

Table S2. EQ-5D-3L Mixed Regression Model: 6MWD (Centered on Mean)

Category	Coef.	SE	z	*P* > z	95% LCI	95% UCI
6MWD_m	0.00055	0.00009	6.05000	0.00000	0.00037	0.00073
Female	−0.07185	0.02570	−2.80000	0.00500	−0.12223	−0.02147
Constant	0.68629	0.01709	40.15000	0.00000	0.65279	0.71979

Abbreviations: 6MWD_m, 6-minute walk distance (centered on mean); coef, coefficient; LCI, lower bound confidence interval; SE, standard error; UCI, upper bound confidence interval.

Table S3. EQ-5D-5L Mixed Regression Model: 6MWD

Category	Coef.	SE	z	*P*>z	95% LCI	95% UCI
6MWD	0.001	0.000	6.070	0.000	0.000	0.001
Constant	0.533	0.036	15.000	0.000	0.463	0.603

Abbreviations: 6MWD, 6-minute walk distance; coef, coefficient; LCI, lower bound confidence interval; SE, standard error; UCI, upper bound confidence interval.

Table S4. EQ-5D-5L Predicted Utility Values by 6MWD

Category	6MWD (m)		
	≤75	>75 to ≤250	>250[a]
EQ-5D-5L utility value	0.55	0.62	0.75

[a]Maximum reported: 678 m.
Abbreviation: 6MWD, 6-minute walk distance.
